# Supplementary material for: Screening for and Disclosure of Domestic Violence during the COVID-19 Pandemic: Results of the PRICOV-19 Cross-Sectional Study in 33 Countries
Source: Int J Environ Res Public Health. 2023 Feb 16;20(4):3519. doi: 10.3390/ijerph20043519 (PMC9964689; doi:10.3390/ijerph20043519)
Supplement: Supplementary file 1 [file ijerph-20-03519-s001.zip › ijerph-2181307-supplementary.pdf]

**Supplementary Materials**:

Table S1. Responses in each country for the domestic violence variables, sampling method, and overall response rate.

Table S2. Results of logistic mixed model analysis of potential predictors for disclosure of DV by the patient to the GP during the COVID-19 pandemic.

Table S3. Results of logistic mixed model analysis of potential predictors for screening for DV by the GP during the COVID-19 pandemic.

**Table S1.** Responses in each country for the domestic violence variables, sampling method, and overall response rate.

| Country | n | Study Invitation Sent to [40]: | Overall Response Rate [40] |
|---|---|---|---|
| Austria | 121 | Random national sample | 28.0% |
| Belgium | 456 | Random national sample with additional convenience sample | 29.7% |
| Bosnia and Herzegovina | 31 | Total population | 5.5% |
| Bulgaria | 89 | Convenience national sample | 94.3% |
| Croatia | 121 | Convenience national sample | 11.7% |
| Czech Republic | 96 | Random national sample from four regions and from list of young practitioners | 22.0% |
| Denmark | 36 | Total population | 1.5% |
| Estonia | 108 | Total population | 13.9% |
| Finland | 89 | Convenience national sample | 15.5% |
| France | 512 | Total population | 2.1% |
| Germany | 247 | Convenience national sample | 15.5% |
| Greece | 58 | Random national sample | 94.0% |
| Hungary | 183 | Convenience national sample | 23.4% |
| Iceland | 28 | Convenience national sample | 23.8% |
| Ireland | 168 | Total population | 12.2% |
| Israel | 49 | Convenience national sample | 21.8% |
| Italy | 141 | Convenience national sample | 25.6% |
| Kosovo * | 64 | Convenience sample in five areas | 73.3% |
| Latvia | 122 | Total population | 9.2% |
| Lithuania | 43 | Convenience national sample | 22.5% |
| Malta | 8 | Total population | 6.5% |
| Moldova | 50 | Convenience sample from two municipalities | 24.2% |
| Netherlands | 154 | Random national sample with additional convenience sample | 18.9% |
| Norway | 126 | Total population | 10.5% |
| Poland | 174 | Convenience national sample | 10.4% |
| Portugal | 190 | Random national sample with additional convenience sample | 22.9% |
| Romania | 91 | Convenience national sample | 25.0% |
| Serbia | 106 | Convenience national sample | 90.0% |
| Slovenia | 171 | Convenience national sample | 19.8% |
| Spain | 254 | Convenience national sample | 77.0% |
| Sweden | 21 | Convenience national sample | 7.2% |
| Switzerland | 79 | Convenience sample | 32.0% |
| Turkey | 109 | Convenience sample | 27.9% |
| TOTAL | 4295 | | 27.8% |

* All references to Kosovo, whether the territory, institutions, or population, in this project shall be understood in full compliance with the United Nations Security Council Resolution 1244 and the ICJ Opinion on the Kosovo declaration of independence, without prejudice to the status of Kosovo.

**Table S2.** Results of logistic mixed model analysis of potential predictors for disclosure of DV by the patient to the GP during the COVID-19 pandemic.

| | Model I | Model II | Model III | Model IV | Model V |
|---|---|---|---|---|---|
| **Fixed Part** | | | | | |
| Position | | 0.961 | | | |
| Years of experience | | **0.011** | 0.161 | | |
| Location of practice | | 0.808 | | | |
| Number of GPs | | **<0.001** | **0.019** | **<0.001** | **<0.001** |
| Patients with/over | | | | | |
| migration background | | 0.246 | | | |
| limited health literacy | | 0.470 | | | |
| financial problems | | 0.570 | | | |
| psychiatric vulnerability | | **<0.001** | **<0.001** | **<0.001** | **<0.001** |
| the age of 70 | | 0.165 | | | |
| chronic diseases | | 0.082 | | | |
| little social support | | 0.064 | | | |
| Increased responsibilities | | | 0.410 | | |
| Happy with task shifting | | | 0.711 | | |
| Preparedness concerning task shifting | | | 0.206 | | |
| Further training for responsibilities needed | | | 0.100 | | |
| Screening for financial problems by GP | | | **<0.001** | **<0.001** | **<0.001** |
| Disclosure of financial problems by patient | | | **<0.001** | **<0.001** | **<0.001** |
| Patients with chronic disorder actively contacted | | | 0.359 | | |
| Patients with chronic condition and in need of follow up actively contacted | | | 0.976 | | |
| Patients with psychological vulnerability actively contacted | | | 0.637 | | |
| Patients with previous DV actively contacted | | | **<0.001** | **<0.001** | **<0.001** |
| Phone protocol used | | | 0.238 | | |
| Phone protocol how often used | | | 0.709 | | |
| GP more involved in actively reaching out to patients | | | 0.670 | | |
| Triage performed by someone else than GP | | | 0.789 | | |
| Triage info present in consultation room | | | 0.557 | | |
| Video consultations before COVID-19 pandemic | | | 0.108 | | |
| Video consultations since COVID-19 pandemic | | | 0.501 | | |
| Walk in hours present | | | 0.144 | | |
| Incidents (scale) : Patients with urgent condition were seen late | | | **0.047** | **0.031** | **0.036** |
| Communication (scale) presence of website, leaflets, answering machine | | | | 0.125 | |
| eWBI | | | | 0.256 | |
| **Random part** | | | | | |
| Country variance (SD) | 0.05 (0.22) | 0.05 (0.21) | 0.03 (0.19) | 0.03 (0.18) | 0.03 (0.18) |
| Practice variance (SD) | 0.41 (0.64) | 0.40 (0.63) | 0.36 (0.60) | 0.36 (0.60) | 0.36 (0.60) |
| ICC in % | 10.5 | 10.5 | 8.9 | 8.5 | 8.5 |
| **Model information** | | | | | |
| AIC | 8452.3 | 8502.1 | 8322.4 | 8047.5 | 8014.9 |
| BIC | 8471.3 | 8743.9 | 8844.4 | 8238.5 | 8167.9 |
| Conditional pseudo-$R^2$ | 10.5 | 14.5 | 21.6 | 20.5 | 20.3 |

Abbreviations: GP = General Practitioner; eWBI = European WellBeing Index; SD = Standard Deviation; ICC = Intra-Class Correlation; AIC = Akaike Information Criterion; BIC = Bayesian Information Criterion.

**Table S3.** Results of logistic mixed model analysis of potential predictors for screening for DV by the GP during the COVID-19 pandemic.

| | Model I | Model II | Model III | Model IV | Model V |
|---|---|---|---|---|---|
| **Fixed part** | | | | | |
| Position | | 0.130 | | | |
| Years of experience | | 0.148 | | | |
| Location of practice | | 0.156 | | | |
| Number of GPs | | 0.066 | | | |
| Patients with/over | | | | | |
| migration background | | 0.103 | | | |
| limited health literacy | | 0.612 | | | |
| financial problems | | 0.165 | | | |
| psychiatric vulnerability | | **<0.001** | **0.005** | **<0.001** | **0.001** |
| the age of 70 | | 0.193 | | | |
| chronic diseases | | 0.306 | | | |
| little social support | | 0.329 | | | |
| Increased responsibilities | | | 0.268 | | |
| Happy with task shifting | | | 0.100 | | |
| Preparedness concerning task shifting | | | 0.150 | | |
| Further training for responsibilities needed | | | 0.232 | | |
| Screening for financial problems by GP | | | **<0.001** | **<0.001** | **<0.001** |
| Disclosure of financial problems by patient | | | **<0.001** | **<0.001** | **<0.001** |
| Patients with chronic disorder actively contacted | | | 0.190 | | |
| Patients with chronic condition and in need of follow up actively contacted | | | 0.183 | | |
| Patients with psychological vulnerability actively contacted | | | 0.062 | | |
| Patients with previous DV actively contacted | | | **<0.001** | **<0.001** | **<0.001** |
| Phone protocol used | | | 0.787 | | |
| Phone protocol how often used | | | 0.265 | | |
| GP more involved in actively reaching out to patients | | | **0.049** | **0.003** | **0.003** |
| Triage performed by someone else than GP | | | 0.611 | | |
| Triage info present in consultation room | | | **0.001** | **<0.001** | **<0.001** |
| Video consultations before COVID-19 pandemic | | | 0.407 | | |
| Video consultations since COVID-19 pandemic | | | 0.267 | | |
| Walk in hours present | | | 0.663 | | |
| Incidents (scale) : Patients with urgent condition were seen late | | | 0.054 | | |
| Communication (scale) presence of website, leaflets, answering machine | | | | **0.033** | **0.034** |
| eWBI | | | | 0.630 | |
| **Random part** | | | | | |
| Country variance (SD) | 0.04 (0.21) | 0.04 (0.20) | 0.03 (0.17) | 0.03 (0.16) | 0.03 (0.16) |
| Practice variance (SD) | 0.40 (0.63) | 0.39 (0.62) | 0.32 (0.56) | 0.32 (0.57) | 0.32 (0.57) |
| ICC in % | 9.9 | 9.8 | 8.0 | 7.4 | 7.4 |
| **Model information** | | | | | |

| | | | | | |
|---|---|---|---|---|---|
| AIC | 8310.6 | 8425.3 | 7771.3 | 7541.3 | 7530.7 |
| BIC | 8328.7 | 8667.2 | 8242.4 | 7741.3 | 7702.5 |
| Conditional pseudo-$R^2$ | 9.9 | 12.3 | 27.2 | 25.7 | 25.7 |

Abbreviations: GP = General Practitioner; eWBI = European WellBeing Index; SD = Standard Deviation; ICC = intra-class correlation; AIC = Akaike Information Criterion; BIC = Bayesian Information Criterion.
